# Cysts and Fistula Arising in Connection with a Persistent Thyro-Glossal Duct

**Published:** 1894-05-12

**Authors:** 


### CYSTS AND FISTULÆ ARISING IN CONNECTION WITH A PERSISTENT THYRO-GLOSSAL DUCT.

The thryro-glossal duct exists in fœtal life as a diverticulum extending from the foramen cæcum on the tongue downwards in the middle line of the neck, in front of the trachæ, and from it is developed the median lobe of the thyroid gland, the lateral portions of the gland being developed from outgrowths from the fourth visceral arch. From this duct several interesting abnormal structures are developed. First, an accessory pyramid to the thyroid gland, extending upwards from the gland in the line of the duct; second, accessory glands in the middle line of the neck, above the thyroid; third, retention mucous cysts in unobiliterated portions of the duct, which burst or are opened, and continue to discharge as median cervical fistulæ; and fourth, sublingual dermoids presenting in the floor of the mouth. The mucous cysts form in what is known as the thyroid part of the duct, *i.e.*, the portion extending downwards below the hyoid bone; the sublingual dermoids in the upper part known as the lingual duct, which extends from the foramen cæcum on the tongue to the hyoid bone. It is with the retention mucous cysts in the thyroid part of the duct that we are concerned in this paper. Our knowledge of the relation of the thyro-glossal duct to these structures is mainly due to Bland Sutton[1] and Marshall.[2] Marshall was able to dissect out the connection of one of these cysts which had formed a median cervical fistula with the persistent duct. The fistula (unlike those due to persistent bronchial clefts) is always pre-preceded by a swelling, which is the mucous retention cyst; this ruptures, or is opened and continues to discharge. They may appear very early in life, but are not usually congenital. In many, a hard cord can be felt running upwards from the cyst or fistula, which is the persistent duct. In order to cure them, not only must the cyst be removed, but the cord must also be dissected out, and sometimes this may be a matter of much difficulty if it runs up above the hyoid bone into the base of the tongue. They always pass behind the hyoid bone, and in some cases the duct becomes obliterated at this level by the developing hyoid bone, but becomes patent again as it ascends to the foramen cæcum. The application of nitrate of silver or the galvano-cautery seldom cures the fistula; nothing short of thorough removal by dissection will effect a cure.

Mr. Arthur Durham[3] in a clinical lecture at Guy's Hospital records some interesting and typical cases. The first was that of a man æt. twenty, in whom the swelling first formed in his neck when he was four years of age. It was lanced and continued to discharge, but at intervals the opening closed and the contents of the cyst recollected and caused him pain. From its upper margin a cord-like structure could be traced, passing upwards to the hyoid bone. At the time the clinical lecture was delivered the cyst had not been operated on, but probably the case is the same as No. 1 of a series of three reported to the Royal Medico-Chirurgical Society[4] at a later date, in which the origin of the fistula in connection with the thyro-glossal duct was shown by the condition found on removal.

Mr. Durham's second case was that of a little girl æt. six. The swelling first appeared at the age of four years, and had been opened and the cyst-wall scraped away, but the discharge continued. A cord-like swelling extended up to the hyoid bone. The fistula and cord were dissected out and the disease was cured.

The third case was a supposed dermoid cyst in the neck, which extended up beyond the hyoid bone. It was successfully removed. Very few particulars are given.

At the meeting of the Royal Medico-Chirurgical Society, already referred to, Mr. R. J. B. Howard described a case in which two cysts formed in connection with a persistent thyro-glossal duct, and Durham stated that Stecheisen had mentioned several cases of multiple cysts, and Schlange[5] also reports a case of median cervical fistula in the operation, for the removal of which he found two fibrous cysts lined with epithelium, one just below, the other just above the hyoid bone, the persistent duct finally opening into the pharynx at the base of the tongue. In order to remove the cysts and duct he resected the hyoid bone, and believes that no harm results from this.

Raymond Johnson in the "Transactions of the Pathological Society"[6] reports two very interesting cases of median cervical fistulæ which were in University College Hospital, and refers to another case which he believes to have been of the same nature.

[1] Tumours, Bland Sutton, 1893, page 308. [2] Marshall, Journal Anat. and Phys., vol. xxvi, p. 94. [3] Clinical Journal, Jan. 3, 1894, p. 150. [4] Lancet, April 14, 1894, p. 934. [5] American Jour. Med. Sciences, Feb., 1894, p. 208. [6] Vol. xli., p. 325.

